# Help me, and I'll help myself (and others). A narrative inquiry into older women's experiences of health and homelessness

**DOI:** 10.3389/fgwh.2026.1740814

**Published:** 2026-10-09

**Authors:** Lara Nixon, Elaine Boyling, Emma Mierau, Rebecca Hughes, Jeuel Ugburo-Shanomi, Martina Kelly

**Affiliations:** Department of Family Medicine, Cumming School of Medicine, University of Calgary, Calgary, AB, Canada

**Keywords:** counter-narratives, experience, gender, health, homelessness, narrative, older women, structural inequity

## Abstract

**Background:**

While women's homelessness is increasingly recognized as a gendered issue, experiences of aging while homeless remain underexplored. This study examined how older women navigate intersections of aging, health, and homelessness, focusing on how these experiences are shaped by structural and social factors.

**Methods:**

Women's lived experiences were explored and interpreted using a narrative approach. Eleven women (aged 57–65 years) took part in two in-depth interviews each, followed by participation in three sequential focus groups. Data were analyzed iteratively informed by Riessman's thematic and structural narrative analysis. Focus groups served as collaborative spaces to provide feedback on initial interpretations and deepen understanding of experiences across social and structural contexts. Co-constructed storylines and composite narratives are presented of participants' re-structured experiences.

**Findings:**

Two main storylines were identified and supported the development of an overarching counter narrative. The first, ‘Hurry up and Wait', describes the tension women felt within bureaucratic systems that appeared to offer assistance but were experienced as burdensome and disempowering. Shelters and temporary housing were depicted as unsafe and detrimental to health, including hampering their ability to maintain family relationships. The second storyline, ‘Making lemonade', reflects women's agency in managing their health and supporting each other. Despite systemic marginalization, women kept meaningful connections with friends, family, and community, which were a vital form of social capital.

**Conclusion:**

In dominant discourses on homelessness, women's experiences are often hidden and seen through stigmatizing views. This study shows how older women competently navigate complex systems and resist this characterization, creating networks and using caregiving roles to provide mutual support. We propose this labor as a previously unrecognized form of social capital through which women who are homeless affirm their worth and support one another.

## 1 Introduction

Experiences of homelessness and housing insecurity are deeply gendered. Women and gender-diverse individuals often follow distinct pathways into homelessness and encounter different forms of support and intervention compared to men (1–3). These populations face disproportionate housing needs and poverty, shaped by systemic disadvantages linked to gender identity—including caregiving responsibilities, wage disparities, and precarious employment (4–9). Intimate partner violence remains a leading cause of women's homelessness in Canada, with approximately 75% of women and gender-diverse individuals experiencing homelessness identifying as survivors of trauma and/or abuse (8, 10).

Women and gender diverse people are more likely to rely on informal networks and survival strategies to meet basic needs, often driven by limited housing options, barriers to assistance, and overwhelmed emergency shelters (4, 8). Although women comprise only 35% of those counted in Canadian homelessness statistics, this figure likely underrepresents the true scale of need due to the prevalence of hidden homelessness among women (4, 11, 12). Hidden homelessness (defined in Box 1) increases vulnerability to exploitation and abuse, and current enumeration strategies—such as Point-in-Time counts—fail to adequately capture the experiences of women and gender-diverse people, leaving them underserved by mainstream systems and policy responses (8).

Box 1 **Definition of homelessness & hidden homelessness (13, 14).**

The Canadian Observatory on Homelessness defines homelessness as: “the situation of an individual, family or community without stable, safe, permanent, appropriate housing, or the immediate prospect, means and ability of acquiring it.” This includes four different groups of people:

1. Unsheltered people who are “absolutely homeless and living on the streets or in places not intended for human habitation.”
2. Emergency sheltered peoples, who are staying in overnight shelters or shelters that offer spaces for those dealing with situations of familial or domestic violence.
3. Provisionally accommodated, which refers to individuals who can find temporary shelter (e.g., transitional homes and second stage housing programs) as well as those who are considered amongst the ‘hidden homeless', which refers to “people who are temporarily staying with friends, relatives, or others because they have nowhere else to live and no immediate prospect of permanent housing.”
4. At risk of homelessness, referring to those who currently have access to housing yet risk losing it due to poverty, precarity, or unsafe housing conditions that fail to satisfy public health and safety standards.

In Canada, older adults experiencing homelessness typically fall into two categories: those who have faced housing instability throughout their lives, and those who become homeless for the first time in later life (15, 16). Long-term homelessness is associated with accelerated aging and early onset of geriatric conditions, including frailty, mobility limitations, and cognitive decline. Individuals experiencing homelessness often present with health profiles comparable to housed individuals 10–20 years older, making age 50 a widely accepted threshold for “older” in homelessness research (15, 17, 18).

In North America, the number of older women experiencing homelessness is growing at an alarming rate (12, 19, 20). Women aged 55 and older now constitute the majority of visibly homeless women in Canada (12). The widening of social inequities since the COVID-19 pandemic has exacerbated this trend, with older women increasingly vulnerable due to cumulative disadvantages over the life course—often compounded by intersectional discrimination related to race, class, ability, sexuality, and age (7, 8, 21–25). Engaging with narratives that reflect these intersectional experiences allows for a deeper understanding of the structural and cultural determinants of homelessness and housing insecurity (26). Moreover, such narratives reveal how women exercise agency—often in micro and relational forms—and co-create informal supports to navigate oppressive systems (27, 28).

Older people experiencing homelessness are neglected in homeless serving systems and they are widely excluded from aged care (15, 29–32). For the past two decades, Canada's dominant homelessness response has centered on scattered-site Housing First models (33). While effective in reducing homelessness among younger populations, these models have largely failed to address the needs of older adults (29, 30). Housing First programs typically offer time-limited, mobile supports with expectations of graduation to independence, and rely heavily on individuals accessing off-site services. Services such as in-home health and psychosocial supports, and transportation, are rarely integrated into these models but widely needed by older adults (15, 23, 31, 32).

Simultaneously, age-based eligibility criteria for seniors' housing and aged care services—often set at 65 years—exclude many older adults experiencing homelessness, particularly those aged 50–64 (29, 34, 35). For women who experience homelessness for the first time in later life, gendered inequalities—such as delayed workforce entry and limited pension contributions—further compound financial insecurity (6, 36). These challenges intersect with policy gaps that restrict access to old age security, pension plans, and seniors' housing, particularly for those nearing but not yet eligible for age-based supports (7, 15). In the absence of appropriate options, and within overburdened and under-resourced systems, older women experiencing housing insecurity face preventable health declines and increased risk of institutionalization (37).

Knowing the systemic barriers faced by older women experiencing homelessness is essential—but insufficient—without a shift in how these experiences are represented and responded to. In contemporary Canadian society, dominant narratives of aging tend to focus on individual responsibility, obscuring the structural determinants of inequality (15, 25, 38). This individualistic framing contributes to victim-blaming discourses that pathologize those impacted by systemic disadvantage, reflecting what Neale (39) describes as a “minimalist response” that perpetuates inequality.

As Stewart and Cloutier argue, patriarchal assumptions embedded in housing policy design presume access to social and material resources, thereby reinforcing barriers for older women seeking secure housing (6). These narratives often stigmatize older women experiencing homelessness, framing them through lenses of dysfunction and personal failure, despite robust evidence of the structural and social determinants that shape their lives (8, 25, 30, 40, 41).

However, women's experiences remain largely invisible to decision-makers across health, housing, and social service sectors (4, 7, 28). This epistemic exclusion undermines the capacity of systems to respond effectively to the realities of older women experiencing homelessness and housing insecurity (42, 43). Without integrating their lived expertise, service planning and policy development risk reproducing the very inequities they aim to address.

Recognizing that prevailing narratives are often constructed about rather than by older women, this study centers the voices of older women with lived experience of homelessness. Our research question was: how do older women experience the intersections of aging, health, and homelessness?

## 2 Methodology

### 2.1 Study design

This was a qualitative study situated within a larger participatory action research (PAR) project, seeking to understand older people's experiences of housing precarity. PAR draws from Paolo Friere's Pedagogy of the Oppressed (44) and social constructivism, which posits that knowledge is co-constructed and shaped through social interaction. From this stance, PAR positions marginalized individuals as experts in their lived experiences and as co-creators of knowledge and action (45, 46).

During participatory meetings, women expressed a desire to share specific experiences related to their health, healthcare, housing, and homelessness. In response, we initiated a focused study using narrative inquiry to explore how participants made meaning of these experiences over time. Narrative inquiry was suited to this aim, as it recognizes stories as complex representations shaped by an individual's experience and social structures, such as gender, social class, and culture (47).

### 2.2 Theoretical orientation

Narrative research comprises a group of qualitative research approaches that use storied accounts from participants to explore a research question. Bleakley distinguishes two broad approaches to narrative: thinking *about* stories, an approach which treats stories as data, supporting a categorical approach to analysis; and thinking *with* stories, a more holistic and integrative approach, which seeks to preserve the integrity and meaning-making of participants (48). Recently, scholars have also advocated a move beyond individually-focused interpretive approaches to draw better attention to intersecting socio-cultural factors that shape narratives, particularly to acknowledge social power relations and to avoid reinforcing oppressive mechanisms (49). This critical approach to narrative inquiry seeks to draw attention to how meaning and identity are storied, constructed and negotiated in relation to social and structural contexts (50, 51). We drew on Crenshaw's conceptualization of intersectionality to explore how power imbalances within systems of care are experienced in relation to multiple co-occurring marginalizing identities (24, 26), including older age, female gender, homelessness, poverty, disability, non-white race, and experiences of abuse. We simultaneously considered how structural stigma (52) and precarity (53), might shape identities.

#### 2.2.1 The research team

Our research team comprises two family physicians (LN, MK), both of whom work clinically with underserved populations and have been working with the Exchange for 6 years. EB is a research staff member with a background in social anthropology and history of medicine and experience supporting PAR projects in collaboration with people dealing with structural marginalization. Three undergraduate students in health sciences (EM), medicine (JO), and sociology (RH) contributed to data collection and engagement. All team members worked with the women throughout the project.

### 2.3 Context

The study was conducted in a large urban center in Western Canada and informed by our prior research to understand relevant provincial and federal systems and structural contexts (54–57). Led by Canadian historian Travis Hay, we worked with government reports and grey literature, select key informants, and academic publications to report upon factors shaping the health and social outcomes of people living with late-life homelessness in Alberta (54). Policy and structures driving these outcomes were explored in four key overlapping areas: the housing and homelessness sector (including income support policy), provincial continuing care, federal and provincial approaches to substance use and harm reduction, and federal Indian Policy and the production of Indigenous homelessness (54). Through this and ongoing work, we have identified how prevailing individualism and ableism largely overlook or blame and stigmatize populations who are made vulnerable to poor health and social outcomes due to structural causes, and as a result rely on social services (54, 57, 58).

### 2.4 Participants and recruitment

Participants were initially recruited through *The Exchange*, a collective of older adults (aged 54–71) all of whom had experience of homelessness in later life (at age 50 or older), who engaged in co-research to expand services that promote health and quality of life (55, 57). We used the Canadian Observatory on Homelessness definition of homelessness (see Box 1).

All women enrolled in the larger study were eligible to participate provided they were residing in the city of study, and self-identified as a woman. Additional participants were recruited from an elder abuse shelter as well as adult shelters and transitional housing settings, using snowball sampling. Snowball sampling was used because the women had both connections and interest in inviting other women with relevant experience to participate. All participants interested in participating in the study were included in the final sample, however, three eligible participants were lost to follow-up due to changes in health and housing circumstances.

### 2.5 Data collection

Data were collected using interviews and sequential focus groups (59). We conducted a baseline semi-structured interview and a follow-up conversational style interview with each participant (*n* = 11 women). Interviews were semi-structured and opened with a general invitation for the participant to share their story, followed by questions exploring their health and homelessness, as well as experiences of accessing supports and housing. All interviews were conducted by EB or LN with the students (EM, RH, JO), lasting 60–150 min. Three sequential focus groups were subsequently held between February-May 2025 with our initial nine participants. These facilitated iterative knowledge exchange, allowing for individual and collective reflection, and deeper exploration of emerging findings. Focus groups lasted one to two hours.

Data were collected between June 2024 and May 2025 for nine participants, and between May and August 2025 for two participants. The two participants recruited between May and August 2025 did not partake in the focus groups; preliminary findings were shared with these participants through one-on-one communication and they were invited to provide verbal or written feedback which informed interpretation.

All data were audio-recorded and transcribed verbatim. After all activities, the research team wrote reflexive field notes, which we shared on a secure platform. Between data collection activities, participants and researchers engaged in ongoing project work, supporting trust-building and relational continuity.

All interactions and processes were guided by a trauma-informed approach (7, 45, 57, 60, 61). Specifically, researchers paid attention to mitigating barriers to inclusion, providing individualized communication and transportation supports, nutritious food, and time for fun and socializing (55, 62). The women were also provided a cash honorarium for participating in each interview or focus group.

### 2.6 Analysis

Our analytic process evolved over the course of the study. Initially, we used coding and thematic organization of interview and focus group transcripts to familiarize ourselves with a large and complex dataset. However, we found that fragmenting participants' accounts into codes and categories often obscured the emotional, relational, and contextual aspects of their experiences. Consequently, we drew on Riessman's thematic and structural narrative analysis, which prioritizes participants' stories as holistic rather than decontextualized categories (47, 63).

This shift moved us from thinking *about* stories to thinking *with* stories (48), focusing on how participants made sense of their experiences of aging, health, and homelessness. We first engaged in close reading of individual narratives, reflecting on each participant's account as a whole and considering the meanings participants attributed to events, relationships, and systems encountered throughout their stories. We then conducted a cross-case analysis to identify shared narrative patterns and recurring understandings across participants attending to context and individual experience.

We also examined how participants structured and told their stories. Consistent with Riessman's structural approach (47), we attended to sequencing, repetition, interruptions, omissions, reflecting on how narrative features revealed participants' meaning-making and the social conditions shaping their experiences. Initially, we attempted to construct timelines to understand pathways into and through housing instability. However, we observed that participants frequently returned to specific experiences, revisiting them with additional detail or emotional reflection, while other periods remained largely unexplored. Rather than interpreting these omissions as gaps in the data, we understood them as narrative choices reflecting participants' agency.

Throughout our analysis, we engaged in team-based reflexive practices, sharing emotional and intellectual responses during analytic meetings. We considered our positionality as female researchers from more privileged backgrounds and asked empathic questions such as: What if this were our mother, grandmother, or sister? These team meeting discussions helped challenge assumptions and enrich our interpretations. The diversity of age and cultural background within our team, including undergraduate students, contributed to a dynamic analytic process, with students often prompting clarification on taken-for-granted concepts. Reflexive notes were documented across a series of analytic meetings.

We developed composite narratives as a representational strategy to communicate our findings. Although narrative analysis often seeks to preserve the integrity of individual stories, composite narratives have been used by narrative researchers as an ethically sensitive representational strategy that communicates shared experiences while protecting participant anonymity (64). Composite narratives are first-person accounts created from multiple participants' experiences and are used in qualitative research to convey shared experiences while protecting participant anonymity, particularly in studies involving vulnerable populations (64–66). This was important for our participants; several were escaping abusive relationships, and for some of the participants, their housing insecurity was unknown to their families. We constructed our composite narratives to include verbatim quotes from multiple participants, which we documented as part of our audit trail.

### 2.7 Ethics statement

This study received ethical approval from the University of Calgary Conjoint Health Research Ethics Board. All participants provided written informed consent prior to study commencement. We employed process consent (67), revisiting consent at each encounter to ensure participants felt comfortable continuing. This approach, originally developed for research with individuals experiencing dementia, was particularly relevant given participants' experiences with complex health and other life challenges.

## 3 Results

### 3.1 Participants

Eleven women (57–65 years) participated in the study. Four of the women's first experience of homelessness occurred at age 50 or older, four between age 40–50, one at age 30, and two as teenagers. The health and housing status of the women at the time of the baseline interviews is included in Table 1. Of the seven staying in shelter, five were staying at a shelter providing for people with experiences of abuse aged ≥55, two were staying in adult homelessness emergency shelters; three were renting studio apartments in transitional housing and one was renting a studio apartment in market housing having just moved from shelter. During the study, both health and housing status were dynamic: three participants moved between different shelters; one was evicted from their transitional housing to shelter; and five participants moved two or more times, including from temporary stays with family. One woman was living with a partner, another's partner was living outside of Canada, and the remaining nine were unpartnered. All the women except one had been married/partnered. All the women were mothers.

**Table 1:** Participant details at baseline interview.

Name	Age	Health^a^	Housing
Venus	50–64	foot problems, diabetes mobility issues, anxiety	shelter
Kathleen	50–64	heart disease, stroke, memory problems, chronic pain, arthritis, mobility issues, falls, stomach problems, anxiety, depression	shelter
Jeanne	50–64	joint problems, depression	shelter
Brigitte	50–64	diabetes, heart failure, memory problems, chronic pain, joint problems, mobility issues, dental issues, stomach problems, bladder problems	shelter
Brenda	50–54	cancer, diabetes, lung problems, memory problems, chronic pain, joint problems, mobility issues, skin problems, foot problems, dental problems, liver problems, anxiety, depression, PTSD, personality disorder	transitional housing
Cheryl	50–64	cancer, high blood pressure, lung problems, head injury, frostbite, dental problems, liver problems, stomach problems, anxiety, depression, PTSD, ADHD	transitional housing
Denise	50–64	cancer, diabetes, memory problems, chronic pain, joint problems, mobility issues, falls, bladder problems, stomach problems, anxiety, depression	shelter
Sarah	65^+^	lung problems, memory problems, head injury, joint problems, skin problems, dental problems, vertigo, anxiety, depression, PTSD, OCD, ADHD	market housing
Nadine	50–64	lung problems, anxiety, PTSD, eating disorder	transitional housing
Danielle	50–64	cancer, memory problems, chronic pain, joint problems, dental problems, osteoporosis, foot problems, anxiety, depression	shelter
Barbara	50–64	lung problems, memory problems, chronic pain, joint problems, mobility issues, falls, dental problems	shelter

a Health: discussion was facilitated using a list of physical and mental health challenges reported by older people experiencing homelessness in Milaney (69).

PTSD, post-traumatic stress disorder; OCD, obsessive compulsive disorder; ADHD, attention deficit hyperactivity disorder.

In terms of their pathways into homelessness, combinations of financial insecurity, relationship breakdown (including intimate partner violence reported by 10 women), and changes in health status, were reported. All the women met the criteria for “multi-morbidity”, reporting two or more co-existing chronic conditions, which is associated with reduced function and quality of life, and increased health service use and premature death (68). Changes in health status and associated disability related to new health events (stroke, new onset diabetes, cancer) and/or cumulative chronic health issues. Several health problems were attributed to, or exacerbated by, past histories of intimate partner and other violence.

### 3.2 Composite narratives of older women's homelessness

To shed light on the lives of older women experiencing homelessness, we present composite narratives that reflect shared experiences across participants (see Boxes 2 and 3). These narratives are followed by an analytic examination of how women navigate structural adversity through two interwoven storylines: “hurry up and wait,” which captures experiences of delay, constraint, and system fragmentation, and “making lemonade,” which reflects everyday forms of resistance, resourcefulness, and care. We examine how these storylines unfold through the women's experiences of: (1) surviving shelters and transitional housing, (2) stabilizing housing and roles, and (3) recovering health and wellness (see Figure 1).

Box 2 **Marilyn's story – “Stuck everywhere”.**

I've worked everything – waitressing, dog grooming, house cleaning, retail. My first husband and I had our own business. He was plumbing and I'd run around, get parts, do the billing. After we divorced, I kept working around my kids - my stepson was really struggling staying in school. Things were really tight but we were way better off on our own—two of my kids got jobs in the oil fields. In 2022, I had a stroke – I was 54. I couldn't work or drive anymore. I'd never been on welfare. I got on basic assistance pretty quick, but it pays nothing. In 2023, my landlord started drinking after his wife died and he raised my rent. I couldn't afford to stay there anymore, emotionally or financially – things got really ugly.

I stayed at the emergency shelter for a few months. It was terrifying—fights, drugs, people overdosing. Or they'd pass out in the bathroom stalls and block the toilets. We had to leave the shelter during the day and it's four realllllllly long blocks with a walker to the library—the only place who let us use the bathroom without buying something. I was always stressing out about wetting my pants.

So, I moved in with my brother—he's the oldest and got our family home. We weren't very close but I was glad to be out of the shelter. Unfortunately, living there was awful—I didn't see it at first, but my sister-in-law is a real bully. She didn't want me leaving any of my things in the kitchen or the bathroom. I wasn't allowed to answer the door. I was literally in a bedroom for two years. Finally, I met a fantastic counselor who explained I was being mentally abused. Luckily, my oldest daughter agreed to let me come stay. I love living with her and my three grand-kids but her ex is scary. They're separated but he hounds her day and night—wants to know where she's been, who she's seeing, what she's doing. She is thinking about moving cities, so I really want to find something on my own. I don't want to slow her down.

It's impossible to find anything affordable though—even studios are over $1200. I get less than $1,000 a month from assistance. I pay $160 a month for meds and $60 for my phone. That leaves $780 for rent, and that's no groceries, transportation, or heat. I found a cheap basement suite with a friend but neither of us has a credit history, or deposit money. My ex took a bunch of credit cards out in my name and never paid. Also, neither of us can use stairs, so even if we could afford it, we couldn't get in or out—what a gong show!

I'm trying for full disability, so I can afford to pay more for rent and get money to move. This means a whole bunch more medical proof. I also need this to get year-round handi-bus and to get on the wait list for affordable and accessible housing—I have a friend that's been waiting for five years. My GP is good when I can get in to see her. She thinks I have Parkinson on top of my stroke. I've had to go for lots of testing—so many expensive taxis. I have been waiting to see a neurologist and hearing specialist for more than a year. My balance got way worse when she tried me on Parkinsons meds. If I was 65, I'd qualify for seniors housing which is cheaper and has an elevator, but I still wouldn't have the money to move. I feel like I'm stuck everywhere. I'm stuck financially. I'm stuck getting help or housing. I'm just stuck. I'm not going anywhere but I've got to keep pushing—keep pushing myself. I want to be there for my grandkids!

Box 3 **Rose's story—“Be a doer”.**

We're all orphans. I never met my mom—she had to put me in care. My son was just a baby when he lost his dad in a car crash. My father lost his mom to a stroke. But he was taken in and raised by another family in our community. That's what our people do when we can—look out for each other.

I've moved around a lot. It was really hard balancing work and caring for my son after his dad died—child services apprehended him when he was five. I'm working really hard to reconnect with my son and our culture. He has some really big challenges with his health. We were living in a place with my ex, and my ex and I weren't getting along very well. Things were tense and my son got really upset, called our landlord. He evicted us all—said he didn't want us fighting and wrecking his place. We lost our damage deposit and a bunch of things that we had put into the place. We didn't damage the place—he was racist. I've had to deal with that a lot—it doesn't matter where you live, it's there. But I don't let that change the way I am. I really try to help people out if I can and “be a doer”. I used to share my food with my neighbour, a mom and her kids—they never had enough eggs and milk.

Sometimes though, I have to protect myself. It can be really hard not helping people I want to. I was living in “low barrier” housing, where they let people drink and smoke drugs. The drug smoke really bothered me and I don't want to breathe that in. I'm finished with drugs—that's all behind me and I've worked really hard to come to terms with all that. There were kids in the building too and the managers weren't doing anything, so I did what I could. I went to building meetings—I made suggestions. There was this young mom who wanted to be my friend, and I tried but I just couldn't—she was using so much. One day she died. She had a little girl.

I ended up at the elder abuse shelter. I was lucky to get a bed—apparently more than 300 a year get turned away. I love the lady I'm sharing a room with, but the common areas can be terrible - yelling and fighting in the kitchen and laundry. Things go missing from your fridge bin. People don't clean up after themselves. You're on your own to deal with this stuff. The staff also put a lot of pressure on you to find a place on your own. I'm lucky to be good on computers. I've put in applications everywhere (and helped a lot of folks). But it's impossible to go see places—I can't take the bus because I've been having more trouble with my balance lately. I don't have anyone to drive me.

I've had troubles with my knees since I was a kid, plus I've been in two car crashes and fell on the ice last winter. I've been waiting four years for surgery. I'm doing everything I can—ice, heat, exercises. I'm trying to get a gym pass so that I can strengthen up my upper body for when I have to be on crutches—it would be so great if these things were more available—surgery, gyms, safe and affordable housing, transportation—you know? Help me help myself.

**Figure 1:**
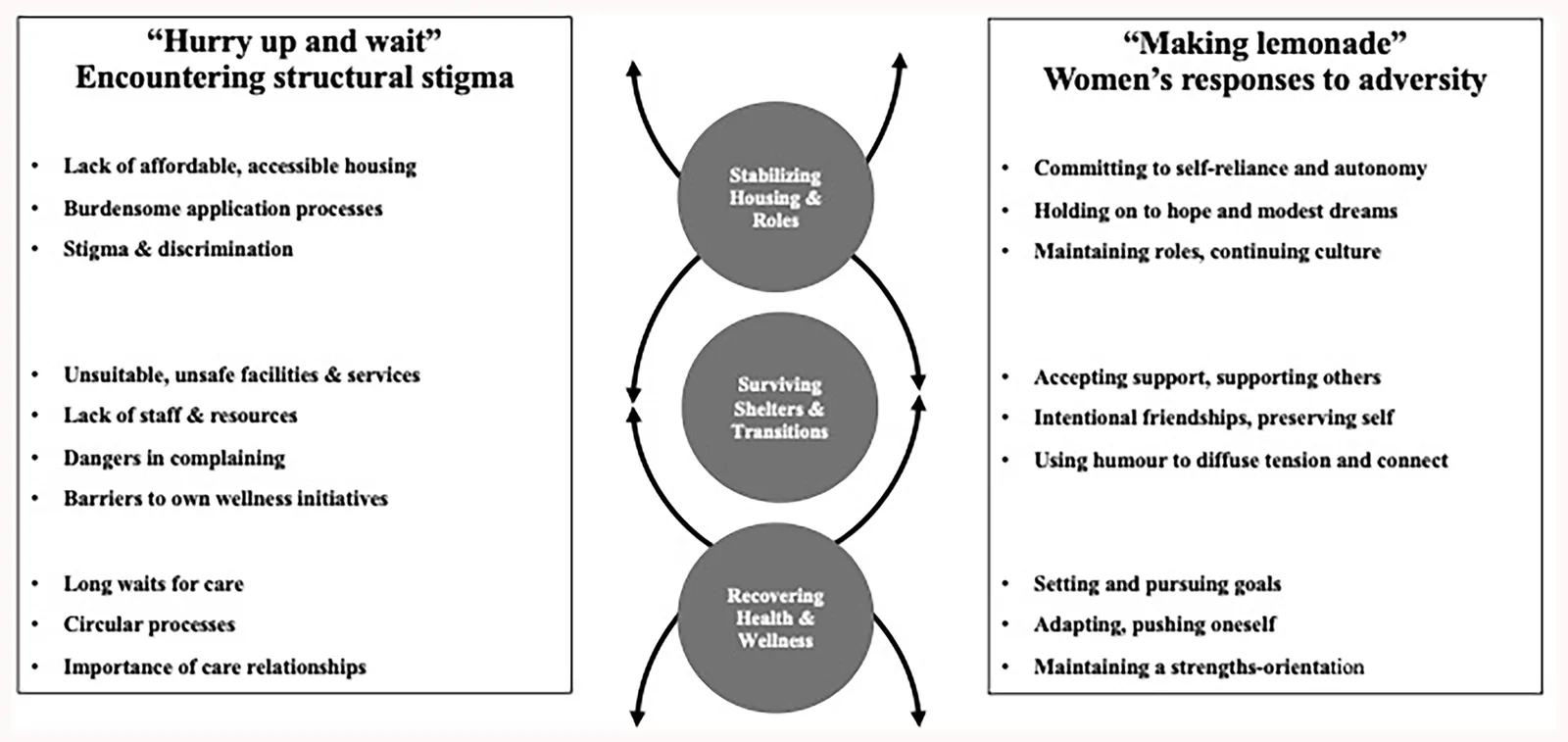
Two interwoven storylines across three siloed systems.

### 3.3 Compounding constraints in siloed systems

Together, these composite narratives illustrate how women's pathways into and through homelessness are shaped by structural stigma related to multiple intersecting identities as women experiencing homelessness, disability, longstanding financial precarity, racism, and abuse. At the same time, they reveal persistent efforts to maintain autonomy, dignity, and care for others. These dynamics are further elaborated through the women's experiences in three siloed systems.

#### 3.3.1 Surviving shelter & transitional housing

Women's experiences in shelters and transitional housing were characterized by a tension between urgency and delay – what participants described as “hurry up and wait.” While conditions often compelled rapid exit, structural barriers to housing prolonged their stays. At the same time, women enacted “making lemonade” through strategies of mutual support, advocacy, and boundary-setting within these environments.

Consistent with the “hurry up and wait” dynamic, participants described shelters as unsafe, unstable, and difficult to inhabit, yet difficult to exit. In addition to drug use, threats of violence, and facility access difficulties as described by Marilyn, participants described unhygienic physical conditions and risk of being barred from shelter use if one filed a complaint:

… a lot of bed bugs, lice… [if staff] even see you with a phone, and they think you're doing that [taking photos of the facilities], they'll confiscate your phone, and you're barred. (Danielle)

Even in a shelter intended for older people fleeing abuse, material and human resource scarcity compromised safety. As Rose described, limited access to essential facilities such as laundry and cooking contributed to distress and conflict, with staff largely unavailable to intervene.

The women also reported difficulties with formal supports in the shelter. Mandatory workshops on nutrition, debt-management, resume writing, and landlord relationships were seen as beneficial. However, some delivery approaches undermined their intent, for example, a male workshop facilitator's use of humor in discussions of intimate parter violence, was distressing:

Laughing and joking…it was supposed to be about abuse. (Kathleen)

Participation in these workshops was mandated by the shelter and repeated participation was required if staying for longer periods. This was seen as unnecessary and even coercive by the women, further reflecting the “hurry up and wait” logic of compliance without progress.

At the same time, participants identified missed opportunities for programming that could reduce conflict and support wellbeing. Activities such as crafts and games nights were highly valued but frequently cancelled due to staff shortages. Two participants, both recognized artists, described offering to contribute their skills as a form of “making lemonade,” but were unable to do so:

[Staff] wouldn't let me have it at the [shelter]. I told them I would teach people there if they would let me. But one worker said yes. Two workers said no. So I didn't get to bring my craftwork. (Brigitte)

Despite these constraints, women actively created supportive micro-environments within shelters, illustrating “making lemonade” through relational care and mutual aid. As Rose and Marilyn reflected, some of the women formed strong bonds in shelter. They searched for housing together, attended health appointments, and exchanged tips on navigating resources. After re-housing, despite distance, some maintained strong friendships that started in shelter:

We talk everyday..and I listen, you know, I give her [Kathleen] support. (Denise)

Having connection with each other, 'someone to talk to’ and laugh with was very important. Connecting through humor, and relieving tensions, functioned as a relational strategy that extended to interactions with staff:

You stand in line, you sign in. And [shelter staff] was behind the glass thing… but I smiled and said, “Hi honey, we're home.” Actually, made her smile. I've never seen her smile. (Barbara)

While transitional housing was often positioned as a step toward stability, participants described conditions that reproduced and in some cases intensified, these challenges. Three participants described gun violence in their transitional housing and were very frightened. One participant confided: I sleep with a bat beside my bed. (Nadine)

As Rose described, other people's drug use in transitional housing brought negative health and emotional impacts, with little intervention by management. These conditions created ongoing tension between self-protection and caring for others—again reflecting the intersection of constraint (hurry up and wait) and response (making lemonade).

Like Rose, attending building meetings and making suggestions, some women expressed more confidence in advocating for change in transitional housing compared to shelter:

Just too much in the hallway, too much drama, drugs, all kinds of crap, you know, and I don't have patience for that shit…I don't like complaining about people, but sometimes you gotta. I mean, when you don't feel safe, you know. I mean, you know, you get in the elevator, and it just reeks of cigarettes or urine. (Cheryl)

Although the women conformed and worked within constraints, they also identified their rights and sometimes, pushed back, advocating for positive change. They carried this “making lemonade” orientation into their efforts to regain housing that aligned with their hopes, including identities as older women.

#### 3.3.2 Stabilizing housing & roles

While participants were expected to rapidly secure independent housing, structural barriers—including affordability, eligibility criteria, and administrative burden—prolonged this process. In response, women enacted persistence, pragmatism, and relational strategies to pursue housing aligned with their needs and identities.

All the women described strong commitment to self-reliance and maintained hopes of finding housing which allowed them to fulfil important roles as carers, friends, and creative people:

I don't want to depend on anybody for anything yet—I'm not ready for that. I still like to do my own thing. I think that's important to me, to be able to do my own thing. It's hard. I got applications out everywhere, to every place. (Brigitte)

Historical and ongoing financial precarity, grief, abuse, and violence were widespread, as are apparent in both Marilyn's and Rose's stories. None of the women voiced expectations of home ownership. All experienced major affordability challenges; many stayed in distressing transitional housing due to lower cost. Some did not pursue financial benefits for fear this could lead their abusive ex-partners to them. In asking about their dream housing, the women wanted to have something “to do”, be it reading, walking in a park, their craft, learning a new language, or traveling. Some hoped for a building that accepted pets. Brenda reported sleeping in a car with her cats for months due to lack of affordable housing that accommodated “[her] babies”. Others aspired to having a second bedroom to accommodate grandchildren for sleepovers, an aging relative, or for craftwork.

The preference for having their own unit often related to difficult past experiences, such as living with strangers who had mental health and addiction issues. Even for those who had a suitable companion to share market housing, structural barriers persisted such as physical inaccessibility, lack of credit histories and insufficient funds for damage deposits and moving. Even when receiving maximum public system income support, benefits were insufficient to be competitive in a private rental market. Eligibility thresholds and benefit structures further reinforced the “hurry up and wait” experience. For example, participants noted that age-based criteria excluded them from seniors' housing prior to age 65, despite clear need.

Applying for income, housing, and transportation supports, was time-pressured and demanding, characterized by ‘hurry up and wait’. Kathleen described being required to phone her case manager every two weeks to stay on a waitlist for accessible subsidized housing. Sometimes the case manager's voice mailbox was full, which put her at risk of losing her spot. Some of the women had good digital literacy, however many others struggled. Seven participants reported benefitting from support of adult children or peers in identifying and navigating online support applications. Some shelter staff were knowledgeable and supportive but were rarely available due to caseloads of 100 people or more.

Additional support from family was available to some, but as both Rose and Marilyn described, these supports had significant limitations related to their children's circumstances. Four of the women held low-wage jobs and used their earnings to support their adult children. Many of the women had grandchildren, and their faces lit up as they spoke about them with pride. However, complex health, social, and learning challenges were common. Some had been separated from their children for many years and were working hard to reconnect, like Rose, as their identity as mothers and grandmothers remained strong.

In addition to prioritizing their roles as carers and forging new friendships, for some women, maintaining longstanding friendships was a key reciprocal role:

But my bestest friend stuck through me through every stupid thing I ever did…she's always been there for me, and I've always been there for her. (Sarah)

Like Rose, some participants drew strength from cultural teachings from childhood that guided their approach to others:

My parents taught me [our cultural ways]. [My mom] used to say, somebody's behavior is not your behavior. Don't do what other people say—it always stuck. So, I always been like that. I don't mind to talk to anybody or walk up to them and say, “Hi, how are you? How's your day?” (Venus)

In their efforts to exit shelter and transitional housing systems, and secure housing that allowed them to pursue important roles, the women demonstrated tenacity, pragmatism, and expertise in navigating complex systems.

#### 3.3.3 Recovering health & wellness

Health challenges were central to participants' experiences of homelessness, both as a consequence of structural inequities and as a barrier to exiting homelessness. These experiences were shaped by the “hurry up and wait” dynamic of delayed access to care, alongside “making lemonade” through active self-management, advocacy, and reframing of adversity.

Similar to how they approached stabilizing their housing, the women took a strong proactive approach to their health. They voiced appreciation for health supports received but long wait times for care were universal and frustrating. Cancellations and rescheduling long-awaited appointments, receiving updates in letters with no options for redress or follow-up, and ‘being on the list’ without being provided clear timelines, all contributed to a sense of powerlessness:

The not knowing…that's the worst…. I have no idea what's going on. (Kathleen)

As Marilyn and Rose described, these waits also had consequences on their ability to function, and to access transportation, housing and funding supports for people with disabilities, leading to compounding vulnerability and a vicious, circular effect characteristic of hurry up and wait.

When they were able to access care, the women were widely positive about their experiences, including primary care and counselling; ten out of 11 women had established relationships with a primary care physician, and four of those 10 were also connected with community nursing. One participant who was receiving outreach nursing support acknowledged this as unusual but essential, following discharge to shelter from hospital for stroke and newly diagnosed diabetes. Three noted the importance of agency to choose their primary care provider and care plans. One participant described how important their relationship with their family doctor had been in regaining wellbeing after experiencing abuse.

I became self-destructive, and I did do a little bit of drugs, but my doctor knew I wasn’t an addict. That was just my medium of self-destructive behavior… I'm lucky that I have my doctor that yeah… he knew me forever. He knew me when I was a super mom … And then he kind of took me under his wing. (Barbara)

Similar to Barbara and Rose, some women alluded to difficult journeys of recovery and re-framed adversity as strength-promoting:

I'm kind of grateful that I'm an addict, because it's taken me on a trajectory where I'm in control of my own life now. I don’t have to be co-dependent. I don't have to be in a relationship with a man to be rescued. (Nadine)

Participants conveyed diverse knowledge of, and commitment to, health promoting behaviors, as seen in Rose's and Marilyn's stories of pushing themselves and advocating for help so they could help themselves. Having access to a healthy diet was a recurrent concern. The women wanted access to fruit, vegetables and meat that ‘didn't come out of a can', and ‘[to shop] beyond the dollar store’. Some women had access to free recreation which they fully embraced. Cold winters and icy sidewalks didn't deter, as Danielle, who uses a walker explains:

Nobody shovels or salts. Part of what gave me this [walker] was all the slips and spills I've taken over the years, and I just can't have another one. No, I will go out, I'm sure. I'll need to, but it will be very little, dainty, diligent steps. I'm taking my time. (Danielle)

In the majority, this “lemonade” positivity was motivated by their family and roles as mothers and grandmothers:

Well grandma's got to be able to keep up with the grandkids, right? So you got to be physically fit and healthy. You know, got to be able to chase them, chase them like crazy (Cheryl)

Despite the multiple adversities that the women faced, and significant health burden, they all expressed a strong will to create a better future for themselves, their loved ones, and community by making lemonade, lots of lemonade.

## 4 Discussion

Older women experiencing homelessness navigated complex intersections of aging, health, and housing insecurity. Rather than facilitating timely access to essential services, their interactions with care systems were often burdensome and disjointed. Bureaucratic processes frequently took precedence over individualized support, with requirements such as contact addresses, credit histories, and assumptions about digital access - and literacy - creating additional barriers. Housing unaffordability, resource scarcity, and service inadequacies contributed to prolonged reliance on homelessness sector supports. Emergency shelters and transitional housing were ill-suited to the physical and psychosocial needs of older women—particularly those with mobility limitations, chronic health conditions, and histories of intimate partner violence or other trauma. For some, engagement with services risked re-traumatization, further complicating their pathways to recovery and stability.

Despite these structural constraints, participants demonstrated considerable agency. They described how they actively “pushed” themselves and supported one another to access health, housing, and social services, and to fulfill caregiving roles. Their resilience, mutual aid, and commitment to self-reliance present a counter narrative to dominant portrayals of older homeless women as dependent, undeserving, or failing to meet gendered expectations of caregiving and domesticity.

### 4.1 Navigating structural barriers

#### 4.1.1 Housing unaffordability & financial vulnerability

Participants identified multiple barriers to securing housing, including financial insecurity, the dual burden of income generation and caregiving, and a lack of age-inclusive housing options. Older women in Canada have been widely shown—especially those who are racialized—to face heightened financial vulnerability due to disproportionate participation in unpaid labor and reliance on public pension supports that are tied to income and contributions (7, 9, 70). Several participants who had worked as homemakers also lacked independent credit histories, which posed significant obstacles to securing rental accommodation and is in keeping with gendered cumulative financial vulnerability identified elsewhere (36, 71). Others expressed fear about disclosing financial information, concerned that it might enable abusive partners to locate them; despite unanimous parliamentary support for a private members bill in 2024, legislation to prevent such coercion failed to become law due to prorogue of Canadian parliament in January 2025 (72, 73).

Alberta, like many Canadian provinces, has not made substantive investments in affordable housing maintenance or construction since the federal government's withdrawal from housing provision in the 1980s and 1990s (74–76). This has resulted in declining stock of affordable housing and escalating waitlists, exacerbated by rising rents and decreasing housing affordability due to unchecked financialization of housing markets (15, 31, 43, 77).

In spite of significant health challenges, some women continued to work or seek employment in low-wage sectors, underscoring the inadequacy of existing income supports as has been confirmed by comparative analysis of social assistance across Canada (78). Importantly, many participants maintained active caregiving roles, supporting adult children and grandchildren, as has been identified by others (5, 27). This aspect of their experience—older women experiencing homelessness as caregivers—remains underexplored in existing literature (2) and offers important insights into the gendered dimensions of aging and housing insecurity.

#### 4.1.2 Barriers to health and wellness compounding financial vulnerability

Participants also faced substantial barriers to healthcare to manage their complex and significant health challenges. Although physician care is publicly funded in Canada, long wait times for specialist medical care had negative impact, not only on women's health, but also on their ability to secure social services (e.g., disability income and transportation supports) and to get on waitlists for accessible, subsidized housing. Mobility challenges limited participants' ability to use public transportation, and while specialized transport services exist for people with mobility issues, these often require proof of high-level disability and impose restrictive conditions (e.g., prohibiting grocery bags or limiting service to winter months); similar barriers to essential wrap-around supports has been identified by others in Canada (7, 35, 79, 80).

Participants' access to primary health care was significantly better and identified as central to their health and wellness. The women highly valued trusting and person-centered relationships with accessible primary providers who respected their autonomy, as has been endorsed by Canadian practice guidelines for care of people with homeless histories (1, 81). Unlike our participant who benefitted from in-shelter nursing support, access to nursing care for people who are unhoused is rare, including post-discharge from hospital, as the emphasis is on home-based care (82, 83).

Delays in securing specialist medical care, including definitive treatment as well as assessments required for income, transportation, and housing, contributed to prolonged stays in homelessness sector services, which in-turn, exacerbated health challenges for many.

#### 4.1.3 Impacts of prolonged stays in shelter and transitional housing

Participants identified multiple challenges with regaining and maintaining their health and wellness during stays in homelessness sector settings such as shelters and transitional housing. Rigid rules and essential resource constraints were badly mismatched to the women's needs and in some cases, experienced as “degrading”, as has been identified by other research in Canada (82, 84). As others have found, mental health and wellness were further undermined by conflict, violence, and others' substance use; in some cases, mental health services were experienced as coercive and undermining rather than supportive, including those that were mandated (1, 28). With so few options, many women faced difficult decisions about sharing accommodation with unfamiliar individuals, which often conflicted with their mental health needs and histories of trauma, similar to findings by others (5, 28). For those who were willing to cohabitate, affordable options such as basement suites were unsuitable due to mobility limitations. Case management, an essential best practice in navigating complex re-housing landscapes for women (1) and older people (79), was largely unavailable to the women due to high client to staff ratios. Some women benefitted from system navigation support from family members and the decisions and actions of all participants reflected strong commitment to self-reliance.

### 4.2 Conforming and resisting: counternarratives of structural competence

Despite facing diverse and significant health challenges, participants demonstrated notable health literacy and proactive engagement with care systems (8). Each woman expressed a clear desire for housing that was safe, private, and calm—conditions they viewed as essential for attending to their own health and supporting the well-being of loved ones (2, 5–7, 85). Many also emphasized the importance of maintaining physical activity and accessing a healthy diet, underscoring their holistic understanding of wellness yet inability to operationalize this knowledge mostly due to limited finances. Permanent rent subsidies could be expected to benefit health through freeing income for these wellness initiatives, as was found in a review by Andermann et al, at a cost that was similar to temporary subsidy programs which did not show benefit (1).

Participants dedicated themselves to conforming to the bureaucratic demands of service systems, including navigating complex application processes and compiling extensive documentation to establish eligibility for income and health supports (4, 28, 86). These demands were frustrating and evoked a sense of precarity (53, 79) but were seen as essential to meet, often driven by their enduring roles as mothers, caregivers, and friends—identities they continued to prioritize despite personal hardship and systemic neglect (5, 27, 28, 87).

In articulating their experiences, the women resisted dominant narratives that portray older adults experiencing homelessness as passive, dependent, or morally deficient. Instead, they framed their circumstances as the result of intersecting structural inequities—such as gendered labor disparities, violence, and policy failures—rather than personal shortcomings. Many critiqued the lack of “respect” embedded in service delivery, emphasizing their lifelong contributions as workers, caregivers, and community members, asserting positive identities (7, 87).

Some participants actively asserted their candidacy for care by negotiating alternative pathways to access services, including switching providers and advocating for personalized care plans (86). When discussing future aspirations, the women voiced modest yet deeply meaningful goals—such as securing stable housing, reconnecting with family, and pursuing creative interests. These expressions of agency highlight their resilience and the enduring importance of dignity, autonomy, and relational well-being in later life.

#### 4.2.1 Relational labor and the production of social capital

By centering the relational experiences shared by participants, we observed not only their resilience but also the tangible outcomes of their sustained efforts to navigate fragmented service systems. The women demonstrated structural competence and relational agency, cultivating and leveraging diverse networks for mutual support and access to care. In a participatory study with older people in Hamilton, Ontario of their experiences of aging with housing precarity, Wyndham identified a similar process of daily negotiations and assertion of agency, as “momentary and productive agency, which allows for the possibility of a hopeful future, despite the gravity of their experiences” (79, p.588). Through these daily interactions, our participants actively built and extended social capital—not only for themselves but also for others in their communities.

Social capital can be understood as the resources and support that people derive from their social relationships, networks of support and community (88), providing a useful framework for understanding these dynamics. Among people experiencing homelessness, social capital has been conceptualized across three interrelated domains: interpersonal relationships (e.g., with family, friends, and service staff), access to formalized services (e.g., food, shelter, healthcare), and the exchange of emotional, practical, and informational support (89). Participants described numerous “bonding” relationships with socially similar individuals, as well as “bridging” relationships with service providers who treated them with dignity and respect. Many spoke of efforts to repair and restore family ties, underscoring the importance of relational continuity. Notably, the women expressed empathy for overwhelmed or burned-out staff while also holding others accountable for perceived abuses of power, advocating not only for themselves but also on behalf of peers even in the face of harsh sanctions for rule-breaking. Similar examples of women experiencing homelessness, defying stereotypes of “needy” or “dysfunctional” were reported by Mayock and Sheridan (28).

These acts of relational labor—frequently invisible and unacknowledged—contributed to the development of individual social capital and could have impact more broadly, through the creation of social infrastructure (90). Social infrastructure, as conceptualized in urban geography by Latham & Layton, includes “people as infrastructure” (90) the informal networks of social and economic cooperation among marginalized individuals in contexts where formal systems are absent or inadequate. The women's relational work functioned as a form of grassroots infrastructure, helping to mitigate the fragmentation and under-resourcing of formal systems. This is in line with other work showing that, despite being underserved, older women are actively navigating systemic gaps, co-creating informal support networks, and engaging in relational labor to survive and support others (5, 7, 8, 27, 91). This is not to say that social capital and mutual aid such as this could be expected to replace formal systems of housing and care. In fact, in recent work, Ayed and Clark caution that homelessness undermines people's ability to practice the reciprocity and exercise autonomy in social relationships central to sustaining social capital (92).

### 4.3 Implications

The intersectionality that is apparent in the women's narratives draws attention to the structural, political and gendered power dynamics their experiences are contextualized within (26) and, further, offers opportunities to look at how the women negotiated oppressive dynamics to find agency, particularly micro-agency and co-construct informal supports to promote this (28). Implications of this study for health care providers include recognizing the gendered nature of older women's experiences of homelessness and system navigation, as well as the critical need for relational approaches grounded in trust-building to promote dialogue and shared decision-making (55, 81, 93, 94). For homelessness organizations and service providers, this study highlights the need for relational continuity to counter the effects of intersecting structural stigmatization and austerity – high client to staff ratios as the women identified, and high rates of staff burnout and turnover (7, 57, 95). Policy is needed to support the provision of trauma-informed, age- and ability-specific services and relational approaches which are expected to have high return on investment for systems (96) and to avoid institutionalization, as the women themselves identified as a priority.

### 4.4 Limitations

Our sample size was intentionally small (*n* = 11) to facilitate in-depth relationship-building and collaborative analysis of participants' situated experiences. While efforts were made to recruit a diverse cohort, individuals identifying as Black, Indigenous, and People of Color were underrepresented relative to their prevalence among the broader population experiencing homelessness. Additionally, none of the participants identified as lesbian, gay, bisexual, transgendered, queer, or two-spirited, despite inclusive recruitment criteria targeting individuals aged 50 and older with lived experience of homelessness.

This limited diversity may be partially attributable to the use of snowball sampling and recruitment pathways linked to service sites, which may inadvertently reproduce the same structural barriers—related to race, gender, and sexuality—that restrict access to services and opportunities for participation in research. Participants themselves identified a range of barriers to inclusive research with people experiencing homelessness. These ranged from relatively straightforward solutions, such as providing translation services for individuals facing language barriers, to more complex challenges, including the need for adequate resources and trauma-informed approaches to support individuals with severe cognitive, psychological, physical, or behavioral health conditions.

Despite these limitations, it is notable that many participants described experiences of hidden homelessness, underscoring the need to broaden definitions and outreach strategies. Throughout the study, the research team recognized and responded to diverse cultural, behavioral, and trauma-related needs within the group. These insights point to opportunities for strengthening participatory action research (PAR) to foster more inclusive practices with older women experiencing homelessness.

Our analytic process developed over the course of the study. Early coding and thematic organization supported familiarization with a large dataset, but we ultimately prioritized narrative interpretation to preserve participants' meaning-making and contextualize experiences within broader social and structural conditions. Different researchers may have privileged alternative narrative features or representations. Our composite narratives were drafted by the research team. This is a potential limitation as it privileges our role as researchers interpreting the data, risking emphasizing some findings over others.

## 5 Conclusion

Older women's experiences of homelessness are often hidden and disregarded, or stigmatized for presumed failure in caregiving and home-making roles. This study shows the competence of older women in navigating complex systems and resisting this characterization. The women engaged in caring for themselves and others, as a form of labor and expertise that goes largely unrecognized. Women's diverse and rich lived expertise of late-life homelessness is central to guiding needed system change.

## Data Availability

The data collected and analyzed in this study are not publicly available due to a confidentiality agreement with the participants and ethical considerations. Initial informed consent and ongoing process consent were undertaken, and participants were assured that their identities and responses will always remain confidential and not go outside of the research team. Requests to access the datasets should be directed to Dr. Lara Nixon, lnixon@ucalgary.ca.
